# The Relationship Between Body Composition and Pathological Response to Neoadjuvant Chemotherapy in Breast Cancer Patients

**DOI:** 10.7759/cureus.61145

**Published:** 2024-05-26

**Authors:** Aysun Isıklar, Ebru Yilmaz, Gul Basaran

**Affiliations:** 1 Internal Medicine, Acıbadem Ataşehir Hospital, Istanbul, TUR; 2 Radiology, Acıbadem Altunizade Hospital, Istanbul, TUR; 3 Oncology, Acıbadem Altunizade Hospital, Istanbul, TUR

**Keywords:** l3 vertebra, pathological response, neoadjuvant chemotherapy, skeletal mass index, operable breast cancer

## Abstract

Background

The pathological response rate in operable breast cancer (BC) patients receiving neoadjuvant chemotherapy (NAC) is postulated to be related to body composition. The success of complete pathological response (pCR) is a known prognostic factor in BC patients treated with NAC. We aimed to accurately measure body composition through BMI and skeletal muscle mass and observe their effects on pCR.

Materials and methods

Patients diagnosed with operable BC who had a positron emission tomography-computed tomography (PET-CT) or chest/abdominal CT taken at the time of diagnosis were retrospectively screened and enrolled in this study. Muscle mass was defined by third lumbar vertebra (L3) level transverse CT images, and data, including weight and height, were collected from the chemotherapy records. All these data were evaluated together with the postoperative pathological results.

Results

Sixty-nine operable BC patients with a median age of 46 (range: 29-72) years were included in the study. In all patients, regardless of sarcopenia, 23% (n = 16) achieved pCR to NAC. The pCR rate was 37.5% (n=6) in sarcopenic patients and 62.5% (n=10) in non-sarcopenic patients (p = 0.530). Overweight (n=4; 25%) and obese (n=2; 12.5%) patients also had a lower pathological response than normal-weight (n=10; 62.5%) BC patients (p=0.261).

Conclusion

Both sarcopenia and obesity independently and synergistically contribute to poorer pathological responses after NAC. Addressing these conditions through tailored interventions, such as nutritional support, exercise programs, and careful monitoring of body composition, could improve treatment outcomes. Further research with larger patient populations and comprehensive body measurements is essential to fully understand these relationships and develop effective strategies to mitigate their impact.

## Introduction

Breast cancer (BC) is the most common type of cancer in women worldwide and the leading cause of cancer-related death among women. One out of every eight women will develop BC in their lifetime [1]. The incidence of BC worldwide is 47.8 per 100,000; in Northern Europe, it is 86.4 per 100,000; in East Asia, it is 43.0 per 100,000; in the United States, it is 90.3 per 100,000; and in Turkey, it is 47.7 per 100,000 [1].

The effectiveness of neoadjuvant chemotherapy (NAC), which is administered before surgical intervention, can be influenced by various patient-specific factors, including body composition. Emerging evidence suggests that conditions such as sarcopenia and obesity negatively impact the pathological response to NAC in BC patients.

Sarcopenia in cancer patients is known as a negative indicator. It has also been reported that obesity is associated with carcinogenesis and cancer progression [2]. Literature reviews on obesity and BC have concluded that obese and overweight BC patients have a higher risk of recurrence and death [2]. The most commonly used measure for obesity is increased BMI, which has been shown to relate to pathological responses from NAC [3]. Over time, it has become clear that BMI is not the sole true indicator of body composition. The term “obesity paradox” describes a condition of being metabolically healthy despite having a high BMI [4]. Therefore, BMI alone does not accurately reflect true body composition, as it does not account for body fat, muscle mass, bone density, and water content [5,6]. Body composition could serve as an imaging biomarker for prognosis and response to treatment in patients with BC [7,8,9].

This study aimed to evaluate body composition as an imaging biomarker to predict the pathological response to NAC in BC patients who were admitted to our center.

## Materials and methods

Study design and patient selection

This is a retrospective, cross-sectional observational, single-center study. It examined individuals with early-stage BC who underwent NAC between February 2017 and December 2022. Patient selection for NAC followed the criteria set by the National Comprehensive Cancer Network (NCCN), which included patients with locally advanced BC and those who could benefit from reducing tumor size before proceeding with conservation therapy.

Participants in this study were chosen based on having had positron emission tomography-computed tomography (PET-CT) or chest/abdominal CT scans at the time of their cancer diagnosis. Skeletal muscle area (SMA), which includes several key muscles, was manually measured by a radiologist blinded to patient outcomes. The measurement focused on the third lumbar vertebra (L3) level of images and encompassed muscles like the rectus abdominis, oblique muscles, transversus abdominis, quadratus lumborum, erector spinae, and psoas. Muscle parameters were measured using IDS7, a digitally signed software component (Sectra AB, Linköping, Sweden).

Various data points were collected from medical records, such as age, weight, height, follow-up duration, menopausal status, tumor characteristics (size, lymph node involvement, subtype), and response to treatment (Residual Cancer Burden (RCB) classification). Patient BMI was categorized according to WHO guidelines: normal (18.5-25 kg/m²), overweight (25-30 kg/m²), and obese (≥ 30 kg/m²). The study also calculated the sarcopenic index (SI) using a formula that considers SMA divided by height at the L3 level. A cutoff value of SI ≤ 38.5 was used to define sarcopenia in women [10].

Statistical analysis

**Figure 1:**
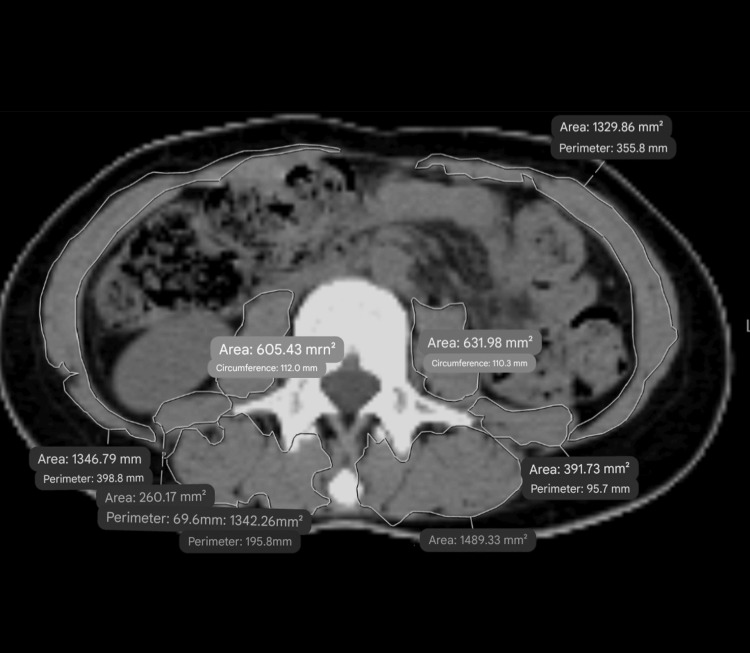
Illustration of the measurement.

SPSS Statistics version 22 (IBM Corp., Armonk, NY, USA) was used for statistical analysis (Figure 1).

While evaluating the research data, descriptive statistical methods (mean, SD, median, frequency, ratio) were used. Additionally, the normal distribution of variables was checked using the Shapiro-Wilk test and box plot graphics.

The Mann-Whitney U test was used to compare variables that did not show a normal distribution according to pCR groups. The Pearson Chi-Square test, Fisher's Exact test, and Fisher-Freeman Halton test were used to compare qualitative data.

Logistic regression analysis was used for the multivariate evaluation of risk factors affecting the pCR response. Additionally, Kaplan-Meier survival analysis and the Log-Rank test were used to evaluate tumor size and disease-free survival, which were determined to be significant based on the pCR. Significance was evaluated at the p<0.05 level.

## Results

Ninety-six operable BC patients who underwent NAC were screened. Of these, 69 whose images were available were included in the study. All enrolled patients were female with a median age of 46 years (range: 29-72). Fifty patients (72.5%) were premenopausal, and 19 (27.5%) were postmenopausal. The median follow-up period was 28 months, during which six patients died. Twenty-two patients were sarcopenic, and 47 were non-sarcopenic. Among the sarcopenic patients, seven were overweight/obese, and 15 were of normal weight (Table 1).

**Table 1:** Basic clinical features of patients. OS: Overall Survival; PFS: Progression-free survival; SI: Sarcopenic Index.

Baseline clinical characteristics of patients	
Age, Median (Min, Max)	46 (29. 72)
Reproductive status	
Premenopausal n (%)	50 (72)
Postmenopausal n (%)	19 (28)
BMI (WHO classification)	
Median (Min, Max)	25 (17.9-43.7)
Normal ≤ 25 n (%)	31 (44.9)
Overweight 25–30 n (%)	23 (33.3)
Obese ≥ 30 n (%)	15 (21.7)
SI (cm^2^/m^2^)	
Median (Min, Max)	43.6 (29-74)
≤ 38.5 (sarcopenic) n (%)	20 (29)
>38.5 (non-sarcopenic) n (%)	49 (71)
BMI of sarcopenic (n=20)	
Sarcopenic with normal BMI n (%)	13 (18.8)
Sarcopenic overweight/obese n (%)	7 (10.1)
Mortalite	6 (8.7)
OS (month) Median (Min, Max)	29.7 (8.9-129.5)
Relapse	13 (18.8)
PFS (month)-Median (Min, Max)	27.7 (8.9-93.7)

The postoperative pathological characteristics of all participants are given in Table 2.

**Table 2:** Postoperative pathologic characteristics of patients. HR: Hormone Receptor; HER2: Human Epidermal Growth Factor Receptor 2; TN: Triple Negative; PCR: Complete Pathologic Response; RCB: Residual Cancer Burden; T: Tumor, N: Lymph Node.

Pathologic characteristics of patients	
Tumor subtype n (%)	
HR positive	56 (81.2)
HER2 positive	9 (13)
TN	4 (5.8)
Stage category n (%)	
Early stage	17 (25)
Advanced stage	52 (75)
Pathologic response n (%)	
pCR (RCB 0)	16 (23.2)
non-pCR (RCB I-III)	53 (76.8)

A total of 16 patients achieved a pCR (RCB 0). The number of sarcopenic patients with pCR was lower (n=7; 44%) compared to the non-sarcopenic group (n=9; 56%) (p=0.359). In the sarcopenic group with pCR, four patients had normal BMI and three were overweight/obese. Overweight (n=4; 25%) and obese (n=2; 12.5%) patients also had a lower pathological response compared to those of normal weight (n=10; 62.5%) (p=0.428). The tumor subtype in the pCR group, regardless of sarcopenia, included 10 HR-positive, four HER2-positive, and two triple negative (TN). The presence of HER2-positive and TN tumor subtypes was higher in the pCR group (25% and 13%, respectively) compared to the non-pCR group (RCB I-III) (9% and 4%). This finding aligns with known chemotherapy sensitivities for HER-2 positive and TN tumors (Table 3).

**Table 3:** Characteristics of patients with pathological complete response (pCR) and non-pathological complete response (non-pCR). T: Tumor; HR: Hormone receptor; HER2: Human epidermal growth factor receptor 2; TN: Triple negative. ^a^Mann-Whitney U test ^b^Pearson Chi-Square test​​​​​​​ ^c^Fisher's Exact test​​​​​​​ ^d^Fisher-Freeman Halton test

Characteristics	pCR (n = 16)	Non-pCR (n = 53)	Total (n = 69)	P-value
Age				
Median (Min, Max)	45.5 (32-65)	45 (29-72)	45 (29-72)	^a^0.293
Tumor size				
Mean±SD;	23.31±17.11	36±18.85	33.06±19.11	^a^0.003**
≤20 m; n (%)	10 (62.5)	12 (22.6)	22 (31.9)	^b^0.003**
>20 mm; n (%)	6 (37)	41 (77.4)	47 (68.1)	
SI				
Mean±SD;	42.02±7.94	44.77±8.53	44.13±8.42	^a^0.181
Sarcopenic; n (%)	6 (37.5)	14 (26.4)	20 (29)	^b^0.530
Non-sarcopenic; n (%)	10 (62.5)	39 (73.6)	49 (71)	
Tumor subtype				
HR positive	10 (62.5)	46 (86.8)	56 (81.2)	^b^0.062
HER2 positive	4 (25)	5 (9.4)	9 (13)	^c^0.197
TN	2 (12.5)	2 (3.8)	4 (5.8)	^c^0.228
Reproductive status				
Premenopausal	13 (81.3)	37 (69.8)	50 (72.5)	^c^0.527
Postmenopausal	3 (18.7)	16 (30.2)	19 (27.5)	
BMI				
Median (Min, Max)	24.8 (20-33.7)	25 (17.9-43.7)	25 (17.9-43.7)	^a^0.442
BMI (WHO classification); n (%)				
Normal weight	10 (62.5)	21 (39.6)	31 (45)	^d^0.261
Overweight	4 (25)	19 (35.8)	23 (33.3)	
Obese	2 (12.5)	13 (24.5)	15 (21.7)	
Stage; n (%)				
Early stage	4 (25)	13 (24.5)	17 (24.6)	^c^1.000
Advanced stage	12 (75)	40 (75.5)	52(75.4)	
Relapse; n (%)				
Yes	0	13 (24.5)	13 (18.8)	^c^0.030*
No	16 (100)	40 (75.5)	56 (81.2)	
Mortality (Ex)				
Yes	0	6 (11.3)	6 (8.7)	^c^0.324
No	16 (100)	47 (88.7)	63 (91.3)	

When evaluating the effects of age, stage, SI, and tumor size on pCR with logistic regression analysis, the model was found significant (F=8.517; p=0.004; p<0.01) with an explanatory coefficient of 76.8%, indicating a good level of model fit. Tumor size was the only significant variable in the model (p<0.01), with a tumor size of 20 mm and above increasing the odds of pCR failure by 5.694 times (95% CI: 1.72-18.89). The effects of age, stage, and SI on pCR failure were not significant (Tables 4-5).

**Table 4:** Logistic regression results of factors affecting pCR. **p<0.01 pCR: Complete Pathologic Response; SI: Sarcopenic Index.

			95% CI	
	P-value	Odds	Lower	Upper
Age	0.140	1.052	0.983	1.125
Stage (Advanced stage)	0.535	0.632	0.148	2.695
SI	0.592	1.023	0.940	1.114
Tumor size (>20 mm)	0.004**	5.694	1.716	18.892

**Table 5:** Disease-free survival analysis according to tumor size. Derived from a Kaplan-Meier analysis.

		N	Relapse (+)	Relapse (-)	Disease-Free Survival Rate	Average Disease-Free Survival Time
Tumor Size	≤20 mm	22	3	19	86.4%	66.81±8.08
	>20 mm	47	10	37	78.7%	67.22±6.23

While no recurrence was detected in 19 cases (86.4%) with a tumor size below 20 mm, three recurrences were observed, indicating an average disease-free survival time of 66.81±8.08 months. In contrast, among 37 cases (78.7%) with a tumor size over 20 mm, 10 recurrences were observed, with an average disease-free survival time of 67.22±6.23 months.

When survival rates according to tumor size were evaluated with the Log Rank test, no statistically significant difference was found between the five-year survival rates (p=0.789; p>0.05).

## Discussion

This study evaluates the relationship between skeletal muscle, body composition, and pathological response to NAC in patients with operable BC. Skeletal muscle and BMI baselines were stratified to assess their impact on NAC. Sarcopenia and overweight/obesity appeared to negatively affect the pathological outcomes, although this was not statistically significant. We used skeletal muscle mass measurements from the L3 spine level on CT scans, which is one of the muscle mass assessment methods most commonly recommended in the literature [10,11,12,13,14]. Randomized studies have shown that pCR to NAC is a predictor of overall survival [15,16]. Additionally, in these studies, baseline skeletal muscle mass values were not related to pCR. Many studies approximate the response to NAC using different methods. Consequently, some of these methods attempt to estimate the effect of NAC using imaging [17,18] and biomarkers [19,20]. However, the exact factors associated with the response to NAC have not yet been identified. The most important reason for this may be the division of BC into molecular subtypes [21].

In our study, the pCR rate was higher in sarcopenic patients and those with a normal BMI, reflecting body composition, but this was not statistically significant. Tumor size has been statistically associated with pCR, which can predict the response to NAC; this suggests that a greater tumor burden may have a negative impact on pCR. This is concordant with previous studies showing that tumor size is associated with the response to NAC [22].

A study of 246 locally advanced BC patients with a median age of 48 years found that skeletal muscle loss and cancer stage were significantly associated with a poor response to NAC. This study showed that skeletal muscle wasting and the clinical stage of cancer are predictive markers for the response to NAC [10]. The effect of obesity was evaluated in a study of 1,169 patients with a median age of 50 years diagnosed with invasive BC. Most of the patients had invasive ductal carcinoma (IDC), were postmenopausal, HR-positive, and HER2-negative. This study demonstrated that the pCR rate was lower in patients with high BMI [12].

Sarcopenia is defined as degenerative skeletal muscle loss, a pathological change resulting from cancer cachexia. Many studies have shown that the presence of sarcopenia has been identified as a negative prognostic marker [11,12]. In support of this data, the number of patients achieving pCR in the sarcopenic group was low in our study.

A case-control study of 67 BC patients who achieved a pCR to NAC was matched with controls who did not achieve pCR. Patients’ skeletal muscle mass was measured from CT images at the L3 level. The response to NAC was not significantly associated with sarcopenia; however, those with sarcopenia tended to have a higher response rate: 18 patients were sarcopenic, of whom 13 (72%) achieved pCR [13]. Another study investigated the prognostic effect of body composition in 248 patients with locally advanced and early-stage BC. Body composition values were obtained by measuring the ratio between visceral adipose tissue and subcutaneous adipose tissue. Included were 198 stage 3 BC patients who underwent NAC from January 2003 to June 2015; 23% achieved pCR, and the median age was 49; 57% were premenopausal. Rates of estrogen receptor (ER)+/HER2-, ER+/HER2+, ER-/HER2+, and TN BC were 57%, 17%, 8%, and 17%, respectively. Fifty percent (n = 37) of patients achieved pCR. The subtypes and BMI were predictive factors for the effects of NAC and prognosis after NAC [14].

A study conducted by the SCAN study group in France from September to October 2017 aimed to determine the prevalence of sarcopenia in metastatic BC patients by assessing CT scan results. Among 139 patients with advanced-stage BC, 41 (29.5%) were sarcopenic, and 57 (41%) were pre-sarcopenic. The study aimed to highlight the importance of sarcopenia in managing metastatic BC, noting that its presence negatively affects prognosis [23].

CT scans are useful tools for evaluating body fat distribution and muscle mass. Litton JK et al. demonstrated in their study that visceral fat has a negative predictive role in pCR to NAC. Specifically, the presence of visceral fat tissue during CT scanning was significantly associated with a lower pCR rate. In the subgroup of patients with pCR, 67% had normal visceral fat, and 92% did not have fatty liver disease, respectively. This evidence confirms that there is no correlation between BMI measurement and the pCR rate found [12]. Similarly, a meta-analysis of eight major trials found no relation between BMI measurement and pCR [2].

As a result, muscle mass or internal fat tissue assessed from CT scan images is more valuable compared to BMI, and more studies are needed on this subject.

The limitation of this study was that the homogeneity of all parameters, including tumor subtype, tumor size, sarcopenia, overweight/obesity, and age range, was unknown. Moreover, the number of sarcopenic and obese patients was low, which may negatively affect the results. The study was retrospectively designed. The measurements were made only by one radiologist, which limits inter-reader correlation.

## Conclusions

Measuring muscle mass from imaging methods (PET-CT or CT) covering the L3 level is easy and applicable. Although the percentage of pCR was lower in sarcopenic and overweight/obese patients, this finding raises the aforementioned “obesity paradox,” and more studies are needed to elucidate this issue.

Both sarcopenia and obesity independently and synergistically contribute to poorer pathological responses after NAC. Addressing these conditions through tailored interventions, such as nutritional support, exercise programs, and careful monitoring of body composition, could improve treatment outcomes. Further research with large patient populations and comprehensive body measurements is essential to fully understand these relationships and develop effective strategies to mitigate their impact.

In summary, sarcopenia and overweight/obesity significantly influence the pathological response to NAC, and addressing these factors is crucial for optimizing cancer treatment outcomes.
